# Pregnancy health in a multi-state U.S. population of systemically underserved patients and their children: PROMISE cohort design and baseline characteristics

**DOI:** 10.1186/s12889-024-18257-8

**Published:** 2024-03-23

**Authors:** Janne Boone-Heinonen, Kristin Lyon-Scott, Rachel Springer, Teresa Schmidt, Kimberly K. Vesco, Anna Booman, Dang Dinh, Stephen P. Fortmann, Byron A. Foster, Jenny Hauschildt, Shuling Liu, Jean O’Malley, Amy Palma, Jonathan M. Snowden, Kalera Stratton, Sarah Tran

**Affiliations:** 1 OHSU-PSU School of Public Health, Oregon Health & Science University, 3181 SW Sam Jackson Park Rd. Mail code: VPT, Portland, OR USA; 2 OHSU School of Medicine, Oregon Health & Science University, 3181 SW Sam Jackson Park Rd, Portland, OR USA; 3 Kaiser Permanente Center for Health Research, 3800 N Interstate Ave, Portland, OR USA; 4 OCHIN, Inc., Portland, OR 1881 SW Naito Pkwy USA

**Keywords:** Gestational weight gain, Body Mass Index, Pregnancy, Child, Retrospective cohort study, Electronic Health Records

## Abstract

**Background:**

Gestational weight gain (GWG) is a routinely monitored aspect of pregnancy health, yet critical gaps remain about optimal GWG in pregnant people from socially marginalized groups, or with pre-pregnancy body mass index (BMI) in the lower or upper extremes. The PROMISE study aims to determine overall and trimester-specific GWG associated with the lowest risk of adverse birth outcomes and detrimental infant and child growth in these underrepresented subgroups. This paper presents methods used to construct the PROMISE cohort using electronic health record data from a network of community-based healthcare organizations and characterize the cohort with respect to baseline characteristics, longitudinal data availability, and GWG.

**Methods:**

We developed an algorithm to identify and date pregnancies based on outpatient clinical data for patients 15 years or older. The cohort included pregnancies delivered in 2005–2020 with gestational age between 20 weeks, 0 days and 42 weeks, 6 days; and with known height and adequate weight measures needed to examine GWG patterns. We linked offspring data from birth records and clinical records. We defined study variables with attention to timing relative to pregnancy and clinical data collection processes. Descriptive analyses characterize the sociodemographic, baseline, and longitudinal data characteristics of the cohort, overall and within BMI categories.

**Results:**

The cohort includes 77,599 pregnancies: 53% had incomes below the federal poverty level, 82% had public insurance, and the largest race and ethnicity groups were Hispanic (56%), non-Hispanic White (23%) and non-Hispanic Black (12%). Pre-pregnancy BMI groups included 2% underweight, 34% normal weight, 31% overweight, and 19%, 8%, and 5% Class I, II, and III obesity. Longitudinal data enable the calculation of trimester-specific GWG; e.g., a median of 2, 4, and 6 valid weight measures were available in the first, second, and third trimesters, respectively. Weekly rate of GWG was 0.00, 0.46, and 0.51 kg per week in the first, second, and third trimesters; differences in GWG between BMI groups were greatest in the second trimester.

**Conclusions:**

The PROMISE cohort enables characterization of GWG patterns and estimation of effects on child growth in underrepresented subgroups, ultimately improving the representativeness of GWG evidence and corresponding guidelines.

**Supplementary Information:**

The online version contains supplementary material available at 10.1186/s12889-024-18257-8.

## Background

Pregnancy is a critical period for the health of birthing parents and their children. Gestational weight gain (GWG) is an easily and routinely monitored aspect of pregnancy health, with higher and lower levels associated with greater risk of adverse pregnancy and birth outcomes [1], maternal postpartum and long-term chronic conditions [2], and offspring health [3]. Accordingly, GWG guidelines from the Institutes of Medicine (IOM, now National Academies of Medicine) [4, 5] draw from extensive evidence and seek to promote healthy GWG, but were last updated in 2009. Critical evidence gaps remain for future revisions of the guidelines, particularly pertaining to pregnant people who have fewer resources, belong to marginalized racial and ethnic groups, or have pre-pregnancy body mass index (BMI) in the lower or upper extremes (underweight, class II or III obesity) [6]. Additionally, evidence of the effects of timing and magnitude of GWG on longer-term child outcomes is relatively scant.

These gaps are difficult to fill with traditional study design: prospective cohorts with longitudinal pregnancy measures tend to underrepresent people with low incomes or other socially or economically marginalized groups, and few data sources provide child outcomes beyond birth. Electronic Health Records (EHR) are a valuable source of repeated weight measures and outcomes in large study populations [7, 8]. Inclusion of large numbers of pregnancies enables investigation of GWG patterns and their associations with health outcomes in subpopulations that are typically understudied due to inadequate numbers of individuals in each subgroup. Further, while many prior pregnancy studies that utilize EHR-derived data include predominately commercially insured patients [9–11], recently developed networks of community-based healthcare organizations (CHCOs) provide rich, longitudinal, clinical data on predominately publicly-insured or uninsured pregnant patients [12]. Yet EHR data pose methodological challenges due to the complexity of clinical data which are not designed for research purposes, particularly in non-integrated care settings.

The PReventing Obesity through healthy Maternal gestational weight gain In the Safety nEt (PROMISE) Study aims to determine overall and trimester-specific GWG associated with the lowest risk of adverse birth outcomes and detrimental infant and child growth in a multi-state U.S. population of CHCO patients. The objectives of this paper are to (1) present the methods and rationale used to (a) construct the PROMISE cohort and (b) develop theory- and data-driven variable definitions with attention to timing relative to GWG and to clinical data collection processes and (2) characterize the cohort with respect to baseline characteristics, longitudinal data availability, and GWG. Throughout this paper, we use neutral weight-related terminology (e.g., high BMI, ≥35 kg/m^2^) where possible, but also recognize the clinical relevance of “obesity classes” and the ongoing ambiguity about the preferred terminology for reducing weight stigma [13]. We recognize that while most pregnant people identify as women, pregnant people can be of any gender, and some are not yet adults. We primarily use the terms “birthing parent” or “pregnant person”, but also use “maternal” to differentiate characteristics of the pregnant person from their offspring.

## Methods

### ADVANCE Clinical Research Network

The PROMISE Study cohort is derived from OCHIN (not an acronym) data from the Accelerating Data Value Across a National Community Health Center Network (ADVANCE) Clinical Research Network [12]. OCHIN is a nonprofit leader in equitable health care innovation and a trusted partner to a growing national provider network. With a centralized EHR system and the largest collection of community health data in the country, OCHIN conducts practice-based research in clinics located in more than 20 states, building patient and provider engagement in research design and implementation at the grassroots. EHR data contain information from the OCHIN Epic® practice management system (e.g., billing and appointments) as well as demographic, utilization, and clinical data from the full OCHIN EHR, much of which has been standardized for research in ADVANCE. The PROMISE study was approved by the Institutional Review Board at Oregon Health & Science University, the lead site for the study.

### Derivation of the PROMISE cohort

In order to study GWG in this unique and understudied patient population, we built upon well-established research using EHR-derived pregnancy cohorts within integrated inpatient and outpatient settings [11, 14, 15]. Because OCHIN data were not linked to inpatient data sources during the study period, our pregnancy algorithm used the extensive outpatient visit data available in OCHIN’s EHR to estimate pregnancy dating needed to define the pregnancy period. In addition, our algorithm defined a cohort for which OCHIN data contain measures, procedures, and diagnoses throughout the study period of interest, enabling longitudinal follow-up of persons regardless of their health insurance status (including lack of insurance or changes in insurance status, common for patients receiving care at CHCOs).

#### Identification of pregnancies

Pregnancies were identified using a process detailed in Supplementary Material 1: Appendix A and summarized here. Two major data sources in the EHR were used: *Pregnancy Episodes of Care* (PE) and *Encounter-based Pregnancy Records* (EPR). An episode of care in the OCHIN EHR is initiated by a provider as a means of providing aggregated information from multiple encounters and data fields for a given clinical condition. A pregnancy-specific episode of care is typically initiated by a medical provider or their support staff at the onset of an individual’s prenatal care. PEs include variables indicating date of last menstrual period (LMP), estimated delivery date (EDD), pregnancy outcome, gestational age (GA) at delivery (when known), and other pregnancy-level information. PEs were used as our primary source of information. The PROMISE team also identified EPRs: pregnancies not associated with a PE. EPRs were defined based on International Classification of Diseases diagnosis codes (ICD-9 and ICD-10) and Current Procedural Terminology (CPT) procedure codes that indicate that the patient was pregnant at the time of an encounter. We used codes that were identified and classified by the Kaiser Permanente (KP) Center for Effectiveness and Safety Research for key attributes including pregnancy outcome, GA, and fetal count. The algorithm was based on work from Hornbrook et al. [16], updated in the KP virtual data warehouse [17] and adapted for OCHIN data by the PROMISE team.

Briefly, in Step 1, a preliminary set of PEs and EPRs were identified among OCHIN records from 1/1/2004 through 1/4/2021, for patients 15 years or older at the time of encounter or pregnancy start. PEs with identical start and end dates were deduplicated, EPRs were assembled from encounter-level data, preliminary start dates were assigned based on outcome date and type (e.g., miscarriage or live birth), and clinical encounters within the preliminary pregnancy start and end dates were extracted. In Step 2, pregnancy start and end dates were refined. Start dates were calculated by subtracting GA at birth, when available, from delivery date; GA at birth is automatically calculated in the OCHIN EHR when both an EDD and delivery date are available. Otherwise, we used the calculated value EDD – 280 days, the latest prior LMP date, or the last recorded encounter diagnosis indicating weeks of gestation, in that order of preference. End dates were refined using birth dates of children who were linked to the pregnant patient within the OCHIN EHR [18], clinician-entered delivery date, or pre- and post-delivery codes, in that order of preference. In Step 3, overlapping or incomplete pregnancy records were removed or consolidated. Self-reported pregnancies noted in a patient’s medical history contain limited patient-reported information (e.g., only pregnancy dates); these pregnancies were excluded if there was no additional information in the EHR. Data sources of the pregnancy records and pregnancy start date and end dates are tabulated in Supplementary Material 1: Appendix A.

#### Inclusion and exclusion criteria

The PROMISE study included pregnancies delivered between 1/1/2005 and 12/31/2020 among OCHIN health network patients 15 years of age or older at pregnancy start. Pregnancies with GA at delivery between 20 weeks, 0 days and 42 weeks, 6 days were retained; current recommendations [19–21] are to induce labor by 41 weeks, though some patients choose to wait until 42 weeks. Therefore, GA longer than 42 weeks, 6 days was considered implausible, likely reflecting inaccurate pregnancy dating, and pregnancies with GA less than 20 weeks were assumed not to be viable. Study inclusion also required availability of ≥ 1 plausible adult height measurement recorded at ≥ 16 years of age and plausible BMI and weight measures required to characterize GWG: ≥ 1 baseline weight measure, ≥ 1 weight measure in the second or third trimester, and ≥ 1 additional weight measure during pregnancy. Plausible height, pre-pregnancy BMI, and weights are defined in Study Variables. Inclusion and exclusion criteria were applied at the pregnancy level, enabling the inclusion of multiple pregnancies per person. We will adjust for within-person correlation in future statistical analyses.

### Data linkages

#### Linkage of parent and child clinical data

At OCHIN, EHR data from parents and children were linked using methods developed and validated as part of a multi-site National Patient-Centered Clinical Research Network (PCORnet) demonstration project [22] and subsequent ADVANCE research [18]. Our linkage methods overcame two data-related challenges in OCHIN data: (a) our data warehouse does not include inpatient data, a common source of maternal-child linkages resulting from hospital-based birth of the child; and (b) as of 2014, Medicaid no longer records household identifiers, which was previously used to link family members in OCHIN clinics located in Oregon [23]. Briefly, explicit, imputed, and fuzzy matches were performed using data available in ADVANCE [18]. Explicit documentation of parent–child relationships included the child ID listed in the parent’s guarantor account information, obstetric claim form, or mother listed in the child’s emergency contact information. Imputed relationships included patient matches on geocoded coordinates for each patient’s last known address, or home phone number. Fuzzy matches compared free-text mother emergency contact demographics against the list of female patients 18 years older in ADVANCE. Parent–child linkages included 66% with explicit linkage, 34% with imputed linkage, and < 1% with fuzzy linkage at the time of the PROMISE Study.

#### Birth record linkage

The subset of EHR pregnancies observed in California, Oregon, and Washington are being linked to birth record data using LinkPlus, a linkage program developed by the CDC [24] and LinkPlus-described procedures including data standardization, calculation of linkage score, and clerical review of matches with uncertain linkage scores. Birth record linkage details are presented in Supplementary Material 1: Appendix B. As of February 2024, Oregon and California linkages were complete, with pending data acquisition from the state of Washington. Linkage rates were 88% and 89% for Oregon and California, respectively.

#### GIS data linkage

ADVANCE patient residential addresses are continuously updated and geocoded, mapped to geographic units (e.g., county, census tract), and linked to US Census and other national data sources [25]. In the ADVANCE population, 75.4% of residential addresses have been geocoded to street address level, 1.6% to street segment, and 23.1% to postal/ZIP code levels.

### Study variables

#### Anthropometry of the birthing person

Maternal weights were extracted from EHR encounters. Plausible weights were defined in two stages. First, weights < 36.3 or > 453.6 kg (< 80 or > 1000 pounds) were discarded (*n* = 323, < 0.01%). Second, we identified pregnancy-specific outliers based on deviation of each measure from temporally adjacent weight measures for the same pregnancy, within each trimester; details are described in Supplementary Material 1: Appendix C. This longitudinal algorithm was adapted from prior work conducted by Sharma and colleagues using pregnancy-related data from the EHR of the Kaiser Permanente Northwest Health Care System [11]. Plausible weights were then used to define baseline and pregnancy weights.

##### Baseline weight

In order to minimize the number of pregnancies excluded due to lack of available baseline weight, we selected the weight closest to pregnancy start date, within 365 days prior to and 97 days after pregnancy start date. Among included pregnancies, the mean duration between the selected weight and pregnancy start date was 7.80 weeks (SD 6.26 weeks); 66,874 (96%) of baseline pregnancy weight measures were taken within 97 days (< 14 weeks) from pregnancy start, with the majority of these measures (83%) from after pregnancy start. Among 19,006 pregnancies with baseline weight measures from the first 97 days of pregnancy that also had pre-pregnancy weight measurements up to 97 days before the pregnancy, the median absolute difference in pregnancy-specific weights was 1.4 kg (25th, 75th percentile: 0.5, 2.5).

When encounter-level baseline weight was unavailable, we used patient-reported pregravid weight (*n* = 7,869, 11%), which was contained in a data field specific for this information in the prenatal vitals section of the EHR. In 38,228 pregnancies in which both sources were available, correlation between encounter-level baseline weight and pregravid weight was 0.99.

##### Height

was defined as the median height among all height measures in the patient’s chart since their 16th birthday. Plausible heights were defined to align with prior literature (48–84 inches [26] [121.9–213.4 cm]).

##### Baseline Body Mass Index (BMI)

Height and baseline weight were used to calculate baseline BMI, used as an approximation of pre-pregnancy BMI. Plausible BMI was defined based on prior studies (12–100 kg/m^2^) [27]. BMI was analyzed as both a continuous and categorical variable [28]: Underweight (< 18.5 kg/m^2^), Normal (18.5 to < 25 kg/m^2^), Overweight (25 to < 30 kg/m^2^), Obesity Class I (30 to < 35 kg/m^2^), Obesity Class II (35 to < 40 kg/m^2^), and Obesity Class III (≥ 40 kg/m^2^).

##### Pregnancy weights

Weights recorded during the pregnancy episode were classified into first (0 to < 14 weeks), second (14 to < 28 weeks), or third (28 weeks-end of pregnancy) trimester.

##### GWG

Total GWG was calculated as last pre-delivery weight minus pre-pregnancy weight, limited to term pregnancies for which the last pre-delivery weight was within 2 weeks of the delivery date. Adherence to IOM guidelines for total GWG was determined based on total GWG and pre-pregnancy BMI category (below, within, or above BMI-specific guidelines), among term pregnancies [5]. Trimester-specific weight gain (total kg, and kg per week) was calculated by fitting a simple linear regression model to measured weights within each trimester for each pregnancy, as described by Abrams & Selvin [29] and applied by others [30]; among pregnancies with two or more observed weights, at least one week apart, in any given trimester. Time coefficients indicate rate of weight gain per week, for each trimester within each pregnancy.

#### Pregnancy and child outcomes

##### Gestational age at delivery (GA)

GA at delivery was calculated within the OCHIN EHR for pregnancies with recorded EDD and delivery date (74%); otherwise, we calculated GA at delivery from start and end dates, defined above (Identification of pregnancies). GA is categorized as preterm birth (GA < 37 completed weeks) and term/postterm (GA (≥ 37 completed weeks). Spontaneous and medically indicated preterm birth are secondary outcomes.

##### Infant size at birth

We extracted birth weight from the PE where available; birth records provide a secondary source within the subset with linked birth records (Oregon, California, Washington deliveries). We defined implausible birth weights based on gestational age- and sex-specific z-scores originally described by Alexander et al. [31] and applied by others [32, 33]: for GA ≥ 37 weeks, < -5 or > 5 SD, and for GA < 37 weeks, < -4 or > 3 SDs calculated within the PROMISE cohort. Size for GA was calculated as an indicator of fetal growth using the reference curve published by Talge et al. [32], enabling calculation of both categorical and continuous measures of birth size, and incorporating clinical estimates of GA, which are more accurate than LMP and readily available in the EHR. Size for GA will be examined in primary analysis as small [< 10th percentile; SGA], appropriate, and large for gestational age [> 90th; LGA]) [31, 34, 35]; and in secondary analysis, using lower SGA [36] or higher LGA [37] cut points that are more clinically meaningful, and as a semi-continuous variable [38]. For comparison with prior literature, we will examine birth weight as a secondary outcome, classified as very low (< 1.5 kg; VLBW), low (< 2.5 kg; LBW), normal, and high (> 4.5 kg; HBW) birth weight.

##### Child anthropometry

We extracted child body weight and length or height, measured on the same day, from clinical encounter records through 1/12/2023. Length (for children < 24 months) and height (for children ≥ 24 months) were recorded in a single field. Longitudinal data availability for child weights and heights for the ADVANCE population has been previously reported [39] and will be presented for the PROMISE cohort in forthcoming outcomes analyses.

We will examine infant growth from birth to 18 months of age, which includes the infant BMI peak (typically at 8–9 months but as late as 17 months) [40, 41]. We will examine growth both in length (cm) and weight (kg), because drivers of weight gain versus length increase differ [42, 43], and explore differences in children born preterm versus full term. For descriptive analysis, we will calculate weight-for-age and length-for-age percentiles and z-scores relative to the World Health Organization (WHO) standard growth curve [44]. We will also explore early life changes in BMI; BMI assesses weight independent of height [45], an indirect measure of adiposity, and it performs better than weight-for-length, even in very young children [46, 47].

To approximate weight status at the critical transition period of school entry, we selected height and weight measured closest to 5 years of age, among measures when children were 4 to < 6 years of age. BMI was calculated and converted to age- and sex-specific BMI percentiles using Centers for Disease Control and Prevention (CDC) 2000 growth curves [48], which is recommended for children 2 years or older [24]. Our primary outcome is continuous BMI z-score, with extended BMI z-score as an alternate outcome that may perform better in children with very high BMI [49]. Our secondary outcome is BMI classification: underweight (< 5th percentile), normal weight (5th to < 85th), overweight (85th to < 95th), obesity (95th to < 20% higher than 95th), and severe obesity (≥ 20% higher than 95th percentile) [50].

#### Covariates

**Fig. 1:**
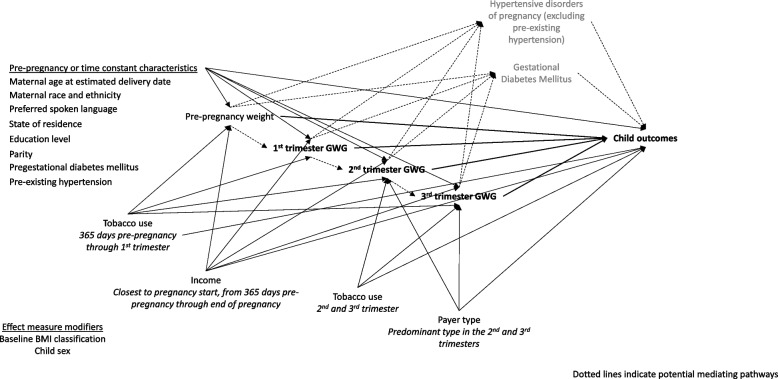
Conceptual framework

We anchored the identification and definition of covariates to our causal framework (Fig. 1).

*Age* of the pregnant person at the EDD was calculated by subtracting the date of birth of the birthing person from the EDD. Clinical care processes are based on age at EDD; for example, designation of pregnancies as having “advanced maternal age” when the birthing person is ≥ 35 years of age at expected delivery.

*Race and ethnicity* are recorded in the patient table in separate fields. In contrast to concerns about missing race and ethnicity information in claims and clinical data [51, 52], CHCOs are federally required to report race and ethnicity, supporting a high degree of completeness in our study population. We combined race and ethnicity into a single variable based on recent guidelines [53], which recognize that race categories typically used in the U.S. are not interpretable for many Hispanic people, resulting in misclassification into white or “other” race or a high level of missingness in the race variable [54]. We created the following race and ethnicity categories, which use the most granular race categories recorded in the EHR: Hispanic, Non-Hispanic [NH] American Indian/Alaska Native, NH Asian, NH Black, NH Native Hawaiian/Other Pacific Islander, NH other/multiple, NH white, unknown. For secondary analyses, more granular race categories will be obtained from birth records among the subset with linked birth record data. We examine race and ethnicity as social variables [53, 55], reflecting a constellation of social, cultural, historical, and interpersonal processes that impact family resources, individual behaviors, experience of psychosocial stress, and biased clinical care delivery that can impact body weight and/or pregnancy and child health.

*Preferred spoken language* is recorded in the patient demographics table (English, Spanish, other, unknown). We examine preferred language as a proxy for cultural and social factors that influence body weight and pregnancy and child health, as well as an indicator of language barriers that can influence clinical care.

*State of residence* was obtained from the patient demographics table and reflects the most recently reported state of residence at the time of data extraction. For descriptive purposes, states were classified as Oregon, Washington, California, and other; these categories reflect the predominant states represented in the PROMISE study population. We examine state of residence as a proxy for geographically-patterned determinants of pregnancy and child health, including variations in clinical practice across states.

*Education at the time of delivery* and *parity* are available in the birth record, among the subset with linked birth records. Education will be categorized as less than high school, high school, some college, college completion or higher and examined as a dimension of socioeconomic position. *Parity* is a known determinant of body weight and pregnancy and child health; it will be categorized as nulliparous or multiparous.

*Income level*, *payer type*, and *smoking status* were obtained from encounter-level data and were defined based on several considerations: the time period(s) of interest, degree of missingness in the target time period, and frequency and process of data collection in CHCOs.

*Income* as a percentage of the federal poverty level (%FPL) is collected by most CHCOs for reimbursement purposes. Household income (USD), state of residence, most recent family size, and year- and region-specific U.S. poverty guidelines are used to calculate %FPL. Given that income destabilizes at around mid-pregnancy in the general population [56], we sought to measure %FPL prior to or early in pregnancy to establish temporal order prior to the exposure of interest. However, defining %FPL within a specific perinatal period is not possible with the existing data collection process: last known %FPL is requested from patients approximately every 6–12 months. Thus, we selected the %FPL value recorded closest to pregnancy start, within 365 days prior to pregnancy through the end of pregnancy. This time frame reduced the number of pregnancies with missing data while, based on preliminary analysis, approximating FPL early in pregnancy. For the purposes of this paper, we classified FPL as ≤ 100%, 101–200%, > 200%, or unknown to characterize the income levels in this cohort.

*Payer type* is recorded at each encounter. We classified payer type as public (Medicaid and Medicare), private, other non-comprehensive insurance (e.g., worker’s compensation, auto, life, farmer’s insurance, private plans specific to dental/vision care, and grant/pilot study coverage), or uninsured. Our goal was to capture insurance and payer status most likely to impact pregnancy health, while recognizing the temporal patterns in insurance throughout pregnancy and that intermittently recorded payer changes are unlikely to reflect actual changes in payer type. Therefore, we selected the predominant payer type throughout the second and third trimesters, defined as the payer type reported at the greatest number of visits. If there was an equal number of visits with multiple payer types, the following hierarchy was applied: Medicaid, Medicare, other public, private. This approach was informed by preliminary analysis showing that payer type changes in early pregnancy, largely due to Medicaid eligibility expansion during pregnancy [57]. Further, we do not expect that first trimester weight gain would influence changes in insurance; that is, second and third trimester payer type is unlikely to be a mediator of the association between GWG and child outcomes.

*Tobacco use* is collected at each encounter as required by EHR-Meaningful use [58]; here, we report use of any tobacco product (smoking or smokeless tobacco). Given well-known changes in tobacco use during pregnancy, potential time-specific effects of tobacco use on weight gain, and preliminary analysis that indicated frequent updates and suggested that changes from current to former user are maintained for the remainder of the pregnancy, we defined tobacco use within two time periods: pre-pregnancy to early pregnancy (365 days prior to pregnancy start through 12 weeks of gestation) and mid-late pregnancy (13 weeks of gestation through the end of pregnancy). In each time period, tobacco use was classified as current (current user at ≥ 1 encounters during the time period), former (no reports of current usage, former user at ≥ 1 encounters during the time period), never (no reports of current or former usage, never or passive/environmental use at ≥ 1 encounters during the time period) user, or unknown. Fewer than 1% reported passive/environmental use, likely reflecting substantial under-reporting; therefore, we are unable to examine passive/environmental use as a separate category.

*Maternal conditions* include pregestational and gestational diabetes mellitus (DM), pre-existing hypertension (HT), and other hypertensive disorders of pregnancy (HDP, including gestational hypertension, preeclampsia, and eclampsia). Pregestational DM was defined as 2 or more encounters with ICD 9 or 10 code indicating DM, or any DM codes on the problem list with onset before pregnancy. GDM was defined as 2 or more GDM ICD 9 or 10 codes in the encounter table during pregnancy or any GDM code on the problem list. HT and other HDP were identified using ICD 9 and 10 codes in encounters or the problem list and evidence of elevated blood pressures in the clinical record. GDM and HDP (excluding HT) are considered mediators in our conceptual framework and will be used in secondary or sensitivity analyses.

*Child sex* (male, female, unknown) is available for PE records or with patient demographic information among children who are also OCHIN patients.

Community-level variables were obtained from linked GIS data. The residential period of interest is during pregnancy; we extracted GIS data corresponding to the residential location recorded closest to the start of pregnancy. Community-level variables include *sociodemographic composition* (racial composition, median income, US Census tract); *modified Retail Food Environment Index* (census tract, CDC); *recreation facilities, fast food restaurants, food stores* (census tract, US Census Business Patterns), and *air quality* (County, Environmental Protection Agency).

### Statistical analysis

We conducted a series of descriptive and longitudinal analyses that inform key factors required to investigate GWG trajectories within pre-pregnancy BMI categories in our unique population.

#### Study population characteristics

We describe the baseline sociodemographic and clinical characteristics of our pregnancy cohort and those excluded from the cohort. Given our objective of estimating effects of GWG trajectories on pregnancy and child outcomes among birthing persons across the spectrum of body size, we also present descriptive characteristics within each pre-pregnancy BMI classification. We focus on the magnitude of group differences rather than statistical testing, given our large sample size.

#### Frequency and timing of GWG data collection

We evaluated the number and timing of pregnancy weight measures, which influence the ability to characterize and analyze GWG trajectories. Specifically, we calculated the average number of weight measures available for each pregnancy, within the total pregnancy period and within trimesters. Data characteristics for children in the OCHIN health system were reported in a previous publication [39].

## Results

### Derivation of the study cohort

**Fig. 2:**
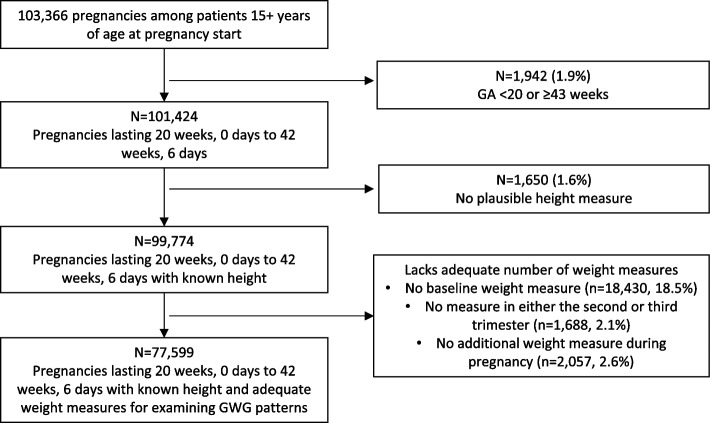
Flow chart

The pregnancy algorithm identified 103,366 pregnancies that started between 4/16/2004 and 7/6/2020 among OCHIN health network patients 15 years of age or older at the start of pregnancy (Fig. 2). Among these, 1,942 pregnancies (1.9%) were excluded due to GA less than 20 weeks or over 43 weeks at delivery (< 20^0^ or > 42^6^ weeks). Exclusions due to lack of required anthropometry data included 1,650 (1.6%) without a known height, and, among the remaining 99,774 pregnancies, 18,430 (18.5%) without a baseline weight measure, 1,688 (2.1%) with no measure in either the second or third trimester, and 2,057 (2.6%) with no additional measure during pregnancy. Thus, the PROMISE cohort includes 77,599 pregnancies lasting 20 to 42 weeks with adequate height and weight measures needed to examine GWG patterns.

### Sociodemographic and baseline characteristics

PROMISE cohort members had a mean age of 27.9 years at delivery, spanning from < 20 years (8.0%) to 40 years and older (3.2%) (Table 1). The largest race and ethnicity group was Hispanic (56.5%), followed by NH white (22.7%) Black (12.1%), and Asian (4.2%). The study population included small proportions (< 1%) but substantial absolute numbers (*n* > 300) of people with NH Native Hawaiian/Pacific Islander or American Indian/Alaska Native race and ethnicity. Over half (53.0%) of the sample had incomes below the poverty level and most had public insurance (82.2%), though with substantial groups who were uninsured (7.6%) or with private insurance (9.5%). The most common preferred spoken languages were English (55.0%) and Spanish (37.8%). Cohort members resided predominately in California (38.5%) and Oregon (27.2%), with the remainder from Washington or other states (6.3 and 27.9%, respectively).

**Table 1:** Characteristics of pregnant individuals, per pregnancy, in the PROMISE Study Population^a^

	**Excluded (*n*** **= 12,667)**	**Included (*n* = 77,599)**
Age at delivery [mean (SD)]	27.0 (6.3)	27.9 (6.1)
Age at delivery [n (%)]		
< 20 years	1427 (11.3)	6208 ( 8.0)
20 to < 25 years	3633 (28.7)	18,915 (24.4)
25 to < 30 years	3329 (26.3)	22,198 (28.6)
30 to < 35 years	2535 (20.0)	17,979 (23.2)
35 to < 40 years	1350 (10.7)	9811 (12.6)
40 + years	393 ( 3.1)	2488 ( 3.2)
Race and ethnicity		
NH-White	2732 (21.6)	17,617 (22.7)
NH-Black	1962 (15.5)	9419 (12.1)
NH-Asian	562 ( 4.4)	3286 ( 4.2)
NH-Native HI/PI	186 ( 1.5)	408 ( 0.5)
NH AI/AN	51 ( 0.4)	349 ( 0.4)
NH-other	111 ( 0.9)	641 ( 0.8)
Hispanic	6620 (52.3)	43,876 (56.5)
Unknown	443 ( 3.5)	2003 ( 2.6)
Preferred spoken language		
English	7061 (55.7)	42,710 (55.0)
Spanish	4378 (34.6)	29,338 (37.8)
Other	1213 ( 9.6)	5511 ( 7.1)
Unknown	15 ( 0.1)	40 ( 0.1)
Region		
OR	3716 (29.3)	21,089 (27.2)
CA	3798 (30.0)	29,885 (38.5)
WA	1556 (12.3)	4900 ( 6.3)
Other	3586 (28.3)	21,677 (27.9)
Unknown	11 ( 0.1)	48 ( 0.1)
Income as a percent of FPL		
0–50%	4537 (35.8)	23,006 (29.6)
> 50–100%	2399 (18.9)	18,062 (23.3)
> 100–200%	1923 (15.3)	15,299 (19.7)
> 200%	549 ( 4.3)	4539 ( 5.8)
Unknown	3249 (25.6)	16,693 (21.5)
Insurance status		
Uninsured	1364 (10.8)	5864 ( 7.6)
Public	9224 (72.8)	63,816 (82.2)
Private	584 ( 4.6)	7392 ( 9.5)
Unknown	1495 (11.8)	527 ( 0.7)
Tobacco use (pre-pregnancy to early pregnancy)		
Current	327 ( 2.6)	6750 ( 8.7)
Former	202 ( 1.6)	6163 ( 7.9)
Never	1111 ( 8.8)	37,646 (48.5)
Unknown	11,027 (87.1)	27,040 (34.8)
Tobacco use (mid-late pregnancy)		
Current	936 ( 7.4)	5663 ( 7.3)
Former	1206 ( 9.5)	9630 (12.4)
Never	5698 (45.0)	51,182 (66.0)
Unknown	4827 (38.1)	11,124 (14.3)
Gestational age at first pregnancy encounter (weeks)		
< 6 weeks	1051 ( 8.3)	20,772 (26.8)
6 to < 9 weeks	570 ( 4.5)	24,451 (31.5)
9 to < 14 weeks	179 ( 1.4)	19,908 (25.7)
14 to < 28 weeks	7609 (60.1)	10,020 (12.9)
28 + weeks	3258 (25.7)	2448 ( 3.2)
Total number of pregnancy encounters in the second and third trimester		
0	1695 (13.4)	11 ( 0.0)
1–2	193 ( 1.5)	4776 ( 6.2)
3–5	3950 (31.2)	9438 (12.2)
6–10	5173 (40.8)	34,240 (44.1)
11–15	1536 (12.1)	25,633 (33.0)
16 +	120 ( 0.9)	3501 ( 4.5)

^a^Data shown are at the pregnancy level and include multiple pregnancies for some patients; pregnancies were among 74,776 individuals total, and 65,179 individuals included in the PROMISE cohort

Compared to included pregnancies, excluded pregnancies were slightly younger, had lower income, were more likely to be uninsured or have unknown insurance type, and were more likely to live in Washington (Table 1). However, the overall racial/ethnic composition was similar between included and excluded pregnancies. Tobacco use is more likely to be unknown in excluded pregnancies. Utilization patterns were consistent with exclusions due to lack of pre-pregnancy or pregnancy weights, as well as the focus on pregnancies for which OCHIN clinics provided prenatal care. That is, the first pregnancy encounter occurred after the first trimester in the vast majority of excluded pregnancies (85.8%); 13.4% of excluded pregnancies had no pregnancy encounters in the second or third trimesters.

PROMISE cohort members span the full spectrum of pre-pregnancy BMI: 2.1% underweight, 33.8% normal weight, 31.3% overweight, 32.7% obesity (18.9% Class I, 8.4% Class II, 5.4% Class III) (Table 2). In general, those with incrementally higher BMI tended to be older, have greater representation of Hispanic and Black patients, have lower income, and were more likely to have public insurance. In two exceptions, Black race and the lowest income level (≤50% FPL) were also more common in those with underweight. Current tobacco use prior to pregnancy was highest in those with underweight or Class III obesity, while tobacco use during pregnancy was highest in those with underweight. State of residence and utilization patterns were similar across BMI categories, although those with obesity were slightly more likely to have an early pregnancy (< 6 weeks) encounter.

**Table 2:** Pre- or early-pregnancy characteristics of included pregnant individuals, per pregnancy, in the PROMISE Study Population, stratified by pre-pregnancy BMI category

	**Underweight (< 18.5)**	**Normal** **(18.5 to < 25)**	**Overweight** **(25 to < 30)**	**Obesity Class I** **(30 to < 35)**	**Obesity Class II** **(35 to < 40)**	**Obesity Class III (40 +)**
N	1658	26,262	24,310	14,673	6536	4160
Age at delivery [mean (SD)]	24.7 (5.4)	26.7 (6.0)	28.3 (6.1)	29.0 (6.1)	28.7 (5.9)	28.9 (5.6)
Age at delivery [%]						
< 20 years	16.8	11.7	7.0	5.1	4.7	2.9
20 to < 25 years	36.9	28.1	22.8	20.8	21.9	21.5
25 to < 30 years	27.0	28.3	28.6	28.2	29.6	31.4
30 to < 35 years	13.9	20.4	24.5	25.1	25.5	26.5
35 to < 40 years	4.4	9.4	13.7	16.2	14.4	14.6
40 + years	1.0	2.1	3.5	4.7	3.9	3.2
Race and ethnicity						
NH-White	30.2	29.0	19.0	17.3	20.3	24.4
NH-Black	17.5	11.4	10.8	12.2	14.6	18.8
NH-Asian	12.8	7.0	3.3	2.0	1.3	0.8
NH-Native HI/PI	<1	0.3	0.4	0.7	1.0	1.1
NH AI/AN	<1	0.4	0.4	0.5	0.7	1.1
NH-other	1.7	0.9	0.6	0.7	1.1	1.2
Hispanic	33.6	47.7	63.1	64.6	58.8	50.7
Unknown	3.4	3.2	2.4	2.0	2.3	2.0
Preferred spoken language						
English	69.2	59.0	47.7	50.1	62.0	73.5
Spanish	16.7	31.4	45.5	44.5	33.9	24.1
Other	13.9	9.5	6.7	5.4	3.9	2.3
Unknown	<1	0.1	0.0	0.0	0.1	0.0
Region						
OR	26.1	27.8	27.1	27.0	26.8	25.6
CA	33.6	38.9	39.4	38.5	37.2	35.1
WA	6.9	6.2	6.1	6.1	6.8	8.0
Other	33.4	27.1	27.3	28.3	29.2	31.2
Unknown	<1	0.1	0.0	0.1	0.0	0.0
Income as a percent of FPL						
0–50%	38.8	30.6	28.0	28.4	30.5	33.1
> 50–100%	18.8	20.0	24.8	26.3	24.9	23.4
> 100–200%	15.1	18.9	20.6	20.4	20.2	18.8
> 200%	5.5	6.8	5.5	5.4	5.1	4.8
Unknown	21.7	23.8	21.1	19.5	19.4	20.0
Insurance status						
Uninsured	6.9	8.1	7.9	7.0	6.6	5.6
Public	83.8	79.3	82.8	84.4	84.6	85.6
Private	8.4	12.0	8.6	7.8	8.1	8.2
Unknown	<1	0.6	0.7	0.7	0.7	0.7
Tobacco use (pre-pregnancy to early pregnancy)						
Current	13.4	9.0	7.1	7.9	10.7	13.6
Former	6.2	8.0	7.1	7.4	9.5	12.3
Never	38.1	44.4	51.5	52.6	49.3	45.1
Unknown	42.3	38.5	34.2	32.1	30.5	29.0
Tobacco use (mid-late pregnancy)						
Current	14.1	8.1	6.0	5.9	8.4	10.0
Former	12.3	12.8	10.7	11.7	14.8	19.0
Never	57.7	63.7	68.8	69.0	64.2	58.6
Unknown	15.9	15.4	14.5	13.4	12.6	12.4
Gestational age at first pregnancy encounter (weeks)						
< 6 weeks	23.3	24.2	26.7	29.0	29.9	32.1
6 to < 9 weeks	29.2	30.5	32.3	31.8	32.5	32.0
9 to < 14 weeks	23.9	26.6	25.9	25.4	23.9	22.6
14 to < 28 weeks	18.0	14.7	12.3	11.5	11.4	10.7
28 + weeks	5.6	4.1	2.7	2.4	2.3	2.5
Total number of pregnancy encounters in the second and third trimester						
0	0.0	0.0	0.0	0.0	0.0	0.0
1–2	7.4	5.7	5.9	6.2	7.1	8.8
3–5	14.2	12.4	11.4	11.8	12.7	14.7
6–10	48.0	46.1	44.0	42.7	42.0	39.5
11–15	27.4	32.5	34.2	33.7	32.5	30.4
16 +	3.0	3.3	4.6	5.5	5.7	6.5

### Longitudinal data characteristics

We examined aspects of longitudinal data availability that impacts the ability to calculate total and trimester-specific GWG using observed weight measures, both overall and within each pre-pregnancy BMI category (Table 3). In the overall cohort, the last observed pregnancy weight measure was recorded in encounters a median of 5 days prior to delivery, and within 2 weeks of delivery for 78.0% of pregnancies. A median of 2, 4, and 6 valid weight measures were available in the first, second, and third trimesters, respectively. The number of available weight measures varied substantially: for example, 10% had only 1 measure while 10% had ≥9 measures in the third trimester. 68.0, 88.2, and 88.0% of the cohort had a sufficient number of weight measures (≥2 measures within any given trimester) needed to calculate rate of weight gain within the first, second, and third trimesters, respectively. In our data, we observed substantial variability due to calculation of weight gain rates based on closely spaced measures; therefore, we also report number and percent of pregnancies with at least 2 measures, at least one week apart, within any given trimester: 63.9, 87.2, and 88.0% of pregnancies in the first, second, and third trimesters, respectively. The availability of pregnancy weights was generally similar across pre-pregnancy BMI categories.

**Table 3:** Longitudinal follow-up of included pregnant individuals in the PROMISE Study Population

		**Maternal pre-pregnancy BMI classification**					
	**Total**	**Underweight**	**Normal weight**	**Overweight**	**Obesity Class I**	**Obesity Class II**	**Obesity Class III**
Total number of pregnancies	77,599	1658	26,262	24,310	14,673	6536	4160
Days between last observed weight measure and delivery date [median (10th, 90th %ile)]	5 (1, 46)	5 (1, 50)	5 (1, 43)	5 (1, 43)	5 (1, 46)	6 (1, 53)	6 (1, 63)
Pregnancies with last observed weight measure within 2 weeks of the delivery date (%)	78.0	75.1	79.4	78.5	77.3	75.6	73.0
Number of valid weight measures [median (10th, 90th %ile)]							
First trimester	2 (0, 5)	2 (0, 4)	2 (0, 4)	2 (0, 5)	2 (0, 5)	2 (0, 5)	2 (0, 5)
Second trimester	4 (1, 6)	3 (1, 5)	3 (1, 5)	4 (1, 6)	4 (1, 6)	4 (1, 6)	4 (1, 6)
Third trimester	6 (1, 9)	5 (1, 9)	6 (1, 9)	6 (1, 9)	6 (1, 9)	6 (1, 9)	5 (0, 9)
Pregnancies with 2 + valid weight measures in each trimester (%)							
First trimester	68.0	60.2	64.7	69.3	70.5	70.4	71.6
Second trimester	88.2	84.3	87.4	89.1	89.0	88.1	86.8
Third trimester	88.0	85.9	88.9	88.6	88.0	86.1	82.5
Pregnancies with 2 + valid weight measures, at least one week apart, in each trimester (%)							
First trimester	63.9	55.9	60.3	65.3	66.5	66.7	67.6
Second trimester	87.2	82.9	86.3	88.2	88.2	87.3	85.8
Third trimester	88.0	85.1	88.3	88.0	87.3	85.5	81.9

### Gestational weight gain

Among term pregnancies with a weight within 2 weeks prior to the delivery date (*n* = 56,503), mean total GWG calculated from observed weights was 11.8 kg (Table 4). Within this subset, total GWG was below IOM/NAM recommendations for 25.7% of pregnancies and above recommendations in 42.6% of pregnancies. Among all pregnancies with ≥2 measures at least one week apart within a given trimester (*n* = 49,569, 67,708, and 67,822, respectively), weekly rate of weight gain was 0.00, 0.46, and 0.51 kg per week in the first, second, and third trimesters. Total GWG and second trimester GWG were incrementally lower with higher pre-pregnancy BMI; this pattern was also reflected in percentages gaining below or above IOM/NAM recommendations. In the first trimester, those with underweight exhibited the greatest weight gain, while average weight loss was observed in those with obesity class I, II, and III. Third trimester GWG was more similar across BMI categories, though with slightly lower GWG with higher BMI.

**Table 4:** Total and trimester-specific GWG calculated from observed weights among included pregnancies [mean (SE) unless otherwise noted]

			**Maternal pre-pregnancy BMI classification**					
	**N**	**Total**	**Underweight**	**Normal weight**	**Overweight**	**Obesity Class I**	**Obesity Class II**	**Obesity Class III**
**Total GWG (kg)**^a^	56,503	11.85 (6.49)	14.80 (5.25)	14.10 (5.48)	11.95 (6.04)	9.93 (6.48)	8.57 (7.03)	6.88 (7.92)
**Total GWG (adherence to IOM/NAM recommendations)**^a^ **(%)**								
< recommendations	56,503	25.7	35.3	30.8	18.6	21.0	30.7	41.8
Within recommendations	56,503	31.7	43.2	36.4	31.2	27.4	26.0	22.0
> recommendations	56,503	42.6	21.5	32.8	50.2	51.6	43.3	36.1
**First trimester (kg/week)**^b^	49,569	0.003 (0.45)	0.16 (0.41)	0.07 (0.41)	0.00 (0.43)	-0.05 (0.46)	-0.08 (0.49)	-0.12 (0.56)
**Second trimester (kg/week)**^b^	67,708	0.46 (0.30)	0.57 (0.26)	0.55 (0.27)	0.48 (0.28)	0.39 (0.29)	0.33 (0.30)	0.25 (0.36)
**Third trimester (kg/week)**^b^	67,822	0.51 (0.34)	0.54 (0.31)	0.55 (0.30)	0.51 (0.32)	0.48 (0.34)	0.46 (0.40)	0.45 (0.48)

^a^Among pregnancies with a weight measure within 2 weeks of delivery that also qualify as full-term (at least 37 weeks)

^b^Requiring 2 or more valid weight measurements, at least 1 week apart

## Discussion

The PROMISE cohort is a unique pregnancy cohort of over 77,000 systemically underserved patients. We leveraged outpatient data from a national network of CHCOs to identify and date pregnancies, then extracted and used longitudinal anthropometric and other clinical measures to create study variables that align with our conceptual framework and clinical data collection processes. Our study population provides substantial numbers of understudied subgroups, including racial and ethnic groups traditionally underrepresented in research, very low-income groups, and uninsured patients. These data also provide extensive longitudinal measures needed to characterize and study GWG across the BMI spectrum, including underweight and obesity classes II and III.

A key contribution of this study is the derivation of the PROMISE cohort – including identification of pregnancies and follow-up in linked children – from CHCO outpatient records. The OCHIN network of CHCOs serves an exceptionally large and diverse patient population, using a data structure that provides more longitudinal detail as compared to most existing pregnancy-research data resources (i.e., not relying only on inpatient data and/or health care claims). However, lack of inpatient data does provide unique methodological challenges for pregnancy research, due to the lack of childbirth-related claims data. Our pregnancy algorithm can inform construction of similar cohorts in other patient populations without integrated hospital data.

Indeed, the PROMISE cohort provides greater representation of lower resourced, more racially and ethnically diverse patients than most existing cohorts. Among PROMISE cohort members, 69% were Hispanic or non-Hispanic Black, 90% were publicly insured or uninsured, and over half had incomes below the federal poverty level. In comparison, pregnancy cohorts derived from EHR data from integrated care organizations include, for example, 9 to 31% Hispanic or non-Hispanic Black, with ≤5% Medicaid patients [11, 14]. Other EHR-derived cohorts such as samples from the Magee Women’s Hospital in Pittsburgh [34] offer different dimensions of diversity (28% Black, 40% public insurance). Prospective cohorts are often higher SES (e.g., 63.3% [59] or 44% [60] college graduate or higher education, 64.8% with incomes 350% FPL or higher [61]), though with notable examples of high representation in single-site studies [62, 63] or national studies that either do not follow children after birth [64] or were recruited prior to the rise in obesity prevalance [65, 66].

A second key contribution is our set of explicit, theory- and data-driven variable definitions with attention to timing relative to the pregnancy-related exposure and clinical data collection processes. EHR data are derived from clinical visits that occur with variable frequency, determined by a complex set of factors including health status, health care access, and personal and structural barriers [7]. EHR data are also influenced by clinical workflow and structure of the EHR platform. As a result, data availability within specific time periods can be sparse or, in cases like “last known FPL”, misleading. This is particularly pertinent for pregnancy research: pregnancy is a period of dynamic clinical, behavioral, and social changes, and factors during specific perinatal time frames have distinct impacts on health of the pregnant person and the offspring. We anchored our study variable definitions in an explicit conceptual framework and adapted the definitions to the realities of data availability and data collection processes in our CHCO context. By including detailed definitions and rationale, we hope these processes can be applied and tested further in other EHR-based study populations.

A third contribution is the quantification of the data needed to measure GWG across the BMI spectrum. The PROMISE cohort has a median of 2, 4, and 6 weight measures in the first, second, and third trimesters, respectively, with similar availability across BMI categories. These data are sufficient for calculating trimester GWG using traditional methods, but also provide a foundation for minimizing study exclusions with modeled data in future studies. These observed and modeled longitudinal data will enable us to fill longstanding and broadly recognized knowledge gaps about GWG in those with Class II and III obesity, as well as underweight. These gaps are largely due to insufficient sample sizes in most studies, requiring the combination of obesity class II and III together, or exclusion of underweight. In the PROMISE cohort, we have the ability to examine GWG and other pregnancy characteristics for the most vulnerable groups: for example, those with high BMI and with fewer resources to support behavioral or clinical needs; and those with underweight, who may have additional risk factors such as tobacco use or food insecurity, and without sufficient resources to overcome them.

### Limitations

We recognize limitations of the PROMISE cohort, stemming primarily from the reliance of EHR data on information recorded at clinical encounters at OCHIN clinics. This issue has several implications for potential biases. First, clinical data availability impacted the selection into the cohort: lack of availability of a baseline weight measure (18.4%) was the largest reason for exclusion, driven by typically sparse clinical visits prior to pregnancy. We minimized this exclusion by expanding the time window within which we accepted baseline weights and by incorporating pregravid weight, which is patient-reported at an initial prenatal care visit. Furthermore, while pregnancies that were included in the PROMISE cohort showed some differences in baseline characteristics compared to those that were excluded, these differences were slight, with minimal differences in age, race and ethnicity, and spoken language. Second, missing data within the defined cohort were notable for some variables, particularly for pre- or early-pregnancy characteristics. We minimized missing data through our conceptually- and data-driven process and will incorporate imputation methods in future analyses. Third, we lacked data from care received outside of the OCHIN network, including specialized or inpatient care. EHR-based algorithms for pregnancy identification typically include inpatient diagnoses [67, 68]; while we conducted extensive exploratory analysis to appropriately identify diabetes mellitus and hypertension prior to and during pregnancy using outpatient data, validity of these measures would be improved with inpatient data. Additionally, patients with comorbidities may be referred to specialized care outside of the OCHIN network. Those referred prior to or early in pregnancy could be excluded from our study population, while those referred later in pregnancy may be lost to follow-up, or potentially misclassified (resulting in, for example, underascertainment of pregnancy hypertension). Lastly, we were unable to ascertain diagnoses or procedures that occurred during hospital admissions, including the childbirth hospitalization (e.g., induction of labor, severe maternal morbidity).

Limitations also include the absence of information on key confounders that are not available in the clinical record, such as diet, infant feeding, or other behavioral or contextual influences; however, we draw from external data sources including birth records and GIS data where possible. We acknowledge that pregnancy and child weights and BMI are indirect estimates of adiposity, with systematic measurement error related to race and ethnicity. Finally, OCHIN clinics share a clinical data system (OCHIN Epic®) but are otherwise independent entities. Most OCHIN clinics are Federally Qualified Health Centers, for which funding is tied to data collection, metrics, and certain types of performance, but there remains variation in clinical policies, processes, characteristics, and norms across clinics.

### Next steps and future directions

The PROMISE cohort will support future research needed to inform future revisions of GWG guidelines, which will synthesize evidence informing optimal ranges of GWG that balance a wide range of maternal and child risks. Next steps include the characterization of GWG trajectories and estimation of their effects on child growth, at birth, in infancy, and at the time of school entry. These planned analyses will provide evidence with representation of socially marginalized populations and adequate sample size across all BMI categories.

This research project provides opportunities for future integration of additional data to improve measurement and confounding adjustment, and it supports investigation of additional determinants or outcomes of GWG. For example, follow-up maternal or child data could be incorporated for additional calendar years as data become available. Linkage of inpatient clinical or claims data would enable more robust characterization of pregnancy outcomes and in-hospital procedures or diagnoses. Incorporation of laboratory and other clinical data and natural language processing methods would support creation and validation of additional complex variables, such as asthma, heart disease, or autoimmune disease. Death certificate data can be linked to ascertain the rare outcomes of fetal or neonatal death, in order to quantify potential selection bias or examine as outcome variables. Within a subsample, primary collection of data on infant feeding, household environment, and other behavioral and environmental variables would enable investigation of behavioral pathways and outcomes that occur outside of the clinical setting.

## Conclusions

The PROMISE cohort enables the estimation of effects of GWG rate and timing on child outcomes in subgroups that lack a robust evidence base needed to form guidelines for pregnancy weight gain: those belonging to socially marginalized racial and ethnic populations, who are uninsured, publicly insured, discontinuously insured, or with low or high BMI. The cohort provides extensive longitudinal weight measures throughout pregnancy and, as shown in a previous publication [39], in the offspring. With these unique data, we will characterize GWG patterns and their estimated effects on child growth, ultimately improving the representation of GWG evidence and corresponding guidelines.

### Supplementary Information

**Supplementary Material 1.**

## Data Availability

Raw data underlying this article were generated from multiple health systems across the OCHIN network; restrictions apply to the availability and re-release of data under organizational agreements. Researchers interested in accessing the study data can find relevant information at https://ochin.org/research*”.*

## Abbreviations

ADVANCE: Accelerating Data Value Across a National Community Health Center Network
BMI: Body Mass Index
CHCO: Community-based Healthcare Organization
CPT: Current Procedural Terminology
EDD: Estimated Delivery Date
EHR: Electronic Health Record
EPR: Encounter-based Pregnancy Records
FPL: Federal Poverty Level
GA: Gestational Age
GIS: Geographic Information System
GWG: Gestational Weight Gain
ICD: International Classification of Diseases
IOM: Institutes of Medicine
KP: Kaiser Permanente
LMP: Last Menstrual Period
NAM: National Academy of Medicine
NH: Non-Hispanic
OCHIN: (Not an acronym)
PE: Pregnancy Episodes of Care
PROMISE: PReventing Obesity through healthy Maternal gestational weight gain In the Safety nEt
