# Supplementary material for: Pregnancy health in a multi-state U.S. population of systemically underserved patients and their children: PROMISE cohort design and baseline characteristics
Source: BMC Public Health. 2024 Mar 23;24:886. doi: 10.1186/s12889-024-18257-8 (PMC10960496; doi:10.1186/s12889-024-18257-8)
Supplement: Supplementary file 1 — Supplementary Material 1. [file 12889_2024_18257_MOESM1_ESM.docx]

**Appendix A**

Identification of pregnancies

Pregnancies were identified from two major sources in the EMR: *Pregnancy Episodes* (**PE**) and *Encounter-based Pregnancy Records* (EPR). PEs are documented in the OCHIN Epic® EHR by medical providers, either in the context of OB care or as abstracted from records outside of the OCHIN network. *PEs* are recorded at the pregnancy level, containing a Pregnancy ID, Patient ID, pregnancy end date, and pregnancy-specific variables, including gestational age (GA) at delivery, last menstrual period (LMP), estimated delivery date (EDD), pregnancy outcome, and other pregnancy variables for current and historical pregnancies. In contrast, *EPRs* are based on codes noted by a medical provider in the context of an outpatient visit, which indicate that the patient was pregnant at the time of the encounter. Specifically, *EPR indicators* included ICD-9 and ICD-10 diagnosis codes and CPT procedure codes that were identified and classified by the Kaiser Permanente Center for Effectiveness and Safety Research (CESR) for key attributes including pregnancy outcome, gestational age, and fetal count ^1,2^ or most steps of pregnancy identification, PEs were considered to have more complete and more reliable information than EPRs, so they were treated as our primary source of information.

EDD is recorded in the OCHIN clinical record and can be updated throughout pregnancy; the most recent EDD recorded is used to calculate gestational age at delivery (delivery date – EDD). EDD is determined from the clinical estimate based on ultrasound as the preferred and most common source, with LMP or other information (e.g. estimated date of conception, uterus size) used as needed. Thus, a large majority of EDD estimates are expected to be based on clinical estimate via ultrasound, although we do not have data on the source of EDD estimate for individual pregnancies.

**Step 1: Identify PEs and EPRs** among OCHIN records dated 1/1/2004 through 1/4/2021, for patients 15 years or older at the time of encounter. First, *PEs* were de-duplicated based on Episode ID; or Patient ID, pregnancy end date, and pregnancy start date as calculated from end date and gestational age at delivery. Second, *pregnancy EPRs* were assembled from encounter level data through four steps: (a) Records that occurred during a *PE*, or within 30 days prior or 60 days after a *PE*, were considered redundant to PE information and ignored. (b) Records indicating a known pregnancy outcome were classified into the same episode if they were less then 60 days apart; among a given series of outcome records the last known outcome date was identified as a preliminary pregnancy end date. (c) Preliminary pregnancy start dates were assigned to EPR-based pregnancies, based upon the preliminary pregnancy end date and the outcome type. Preliminary pregnancy start dates were assigned 340 days prior for live and still births, 100 days prior for miscarriages, and 50 days prior for ectopic or molar pregnancies; these bounds were intended to be inclusive, to be used to identify all pregnancy EPRs associated with the pregnancy. (d) Clinical encounters with dates between the preliminary pregnancy start and end dates were extracted. Finally, pregnancies and associated variables and encounters were combined into a preliminary table of pregnancies.

**Step 2: Refine pregnancy start and end dates**. Start dates were refined for all pregnancies, using the best information available. For *PEs*, GA was subtracted from delivery date to calculate the start date of the pregnancy. For all EPR-based pregnancies, start dates were defined by subtracting 280 days from EDD, latest prior LMP date, or the last recorded encounter diagnosis indicating a GA (e.g., Z3A.08 for 8 weeks gestation of pregnancy), in that order of preference.

End dates were refined by using birth dates of children who were linked to the pregnant patient within the OCHIN EMR.^3^ When the parent was linked to multiple children with differing birthdates, we selected the birthdate closest to the clinician-entered delivery date (when available), or the latest birth date within 300 days of the refined pregnancy start date. When a linked child’s birth date was not available, we used the clinician-entered delivery date or else pre- and post-delivery codes (e.g., Z37.0 for single live birth; O92.70 for unspecified disorders of lactation). Among those with both birthdate and clinician-entered delivery dates, dates matched for 80% of pregnancies, and were 2 or fewer days apart for 96% of pregnancies. When pregnancies contained multiple pre- and post-delivery codes, those occurring within 30 days of each other were grouped, the last pre-delivery date and the first post-delivery date were identified. When both pre- and post-delivery codes were present, the midpoint was used as the pregnancy end date. Otherwise, the last pre-delivery date was used, followed by the first post-delivery date. Pregnancies without a refined start date or end date were dropped from the study.

**Step 3: Remove or consolidate overlapping pregnancy records**. For overlapping pregnancy records with the same end date but different start dates, information from the clinician-entered pregnancy episode was retained preferentially. For those with the same start date but different end dates, the record with a child birthdate linkage was retained preferentially, followed by information from the clinician-entered pregnancy episode. For pregnancies with different start *and* end dates, pregnancies were retained and consolidated if the differences between start and end dates were <=2 days. Other pregnancies with overlapping dates were dropped from the study, as their start and end date estimates were considered unreliable.

**Table A1**. Pregnancy information data sources (n=77,691 pregnancies)

|  | **# pregnancies (% pregnancies)** |
| --- | --- |
| **Final Pregnancy Record Source** |  |
| Clinician-entered episode ID | 73,522 (94.63) |
| Procedure or Diagnosis record indicating a pregnancy outcome | 4,169 (5.37) |
|  |  |
| **Pregnancy Start Date Source*** |  |
| Clinician-entered episode information | 73,522 (94.63) |
| EDD | 3,876 (4.99) |
| LMP | 263 (0.34) |
| Record indicating weeks gestation | 30 (0.04) |
|  |  |
| **Pregnancy End Date Source*** |  |
| Child’s birth date | 29,969 (38.57) |
| Clinician-entered episode information | 47,216 (60.77) |
| Pre-delivery or immediate post-partum record | 506 (0.65) |

**Appendix B**

Birth record linkage procedure

Following data standardization (e.g., birth dates in YYYYMMDD format), linkage requires the specification of ‘blocking’ variables (e.g., last name, birth date), one of which must match exactly for LinkPlus to calculate a linkage score, and ‘matching’ variables (e.g., first and middle name), which are scored along with blocking variables to determine the likelihood of a true linkage. As recommended by LinkPlus, pairs with linkage score <7 are rejected as non-matches. We then examine the score distribution of remaining linkages to determine appropriate linkage-specific upper (true match), lower (false match), and uncertain (clerical review) cut-points. Pairs in the uncertain range are assigned match status by consensus after double clerical review.

We conduct birth record linkages in two stages. For children whose EHR records could be linked to an EHR pregnancy, we include the child’s last name and birth date along with the parent’s in our set of blocking variables. For EHR pregnancies that do not link to a child’s EHR records, we can only use the parent’s last name and date of birth as blocking variables. Matching variables in both stages include first name, middle name, and child sex. We also consider parents’ maiden names and address information as identifying variables for use during clerical review. Clerical review of records is organized by different ‘classes’ of linkages, as defined in LinkPlus by which subsets of variables did and did not match identically.

**Appendix C**

Pregnancy weight measure screening algorithm

For weight measurements with *both* a previous and subsequent measurement (either within the same trimester or across only one trimester boundary), we calculated four quantities: 1) absolute change from previous measure, 2) weekly change from the previous measure, 3) absolute change to subsequent measure, and 4) weekly change to subsequent measure. Then, observations exceeding cutoffs for weekly and absolute change from previous and to next with a sign-reversal (i.e. large increase followed by large decrease, or vice versa) are dropped as outliers.

For instances with either no valid previous or no valid next comparison measure, only two quantities are computed: 1) absolute change from either previous or to next and 2) weekly change from either previous or to next. For these measures with only one comparison, if both absolute and weekly changes exceed modified cutoffs (a fixed multiple of the original cutoff value—1.5-2x, depending on the percentile setting), the measure is dropped. The steps described above are repeated in an iterative process until no more outliers can be flagged for removal.

1. Hornbrook MC, Whitlock EP, Berg CJ, et al. Development of an algorithm to identify pregnancy episodes in an integrated health care delivery system. *Health Serv Res*. Apr 2007;42(2):908-27. doi:10.1111/j.1475-6773.2006.00635.x

2. CESR Virtual Data Warehouse. March, 2023, <http://kpco-ihr.org/about-cap-data.html>

3. Angier H, Giebultowicz S, Kaufmann J, et al. Creation of a linked cohort of children and their parents in a large, national electronic health record dataset. *Medicine (Baltimore)*. Aug 13 2021;100(32):e26950. doi:10.1097/MD.0000000000026950
